# A human stem cell-derived neuronal model of morphine exposure reflects brain dysregulation in opioid use disorder: Transcriptomic and epigenetic characterization of postmortem-derived iPSC neurons

**DOI:** 10.3389/fpsyt.2023.1070556

**Published:** 2023-02-16

**Authors:** Emily F. Mendez, Sandra L. Grimm, Laura Stertz, Damian Gorski, Sai V. Movva, Katherine Najera, Karla Moriel, Thomas D. Meyer, Gabriel R. Fries, Cristian Coarfa, Consuelo Walss-Bass

**Affiliations:** ^1^Louis A. Faillace, MD, Department of Psychiatry and Behavioral Sciences, McGovern Medical School, University of Texas Health Science Center at Houston, Houston, TX, United States; ^2^Dan L. Duncan Comprehensive Cancer Center, Baylor College of Medicine, Houston, TX, United States; ^3^Department of Molecular and Cellular Biology, Baylor College of Medicine, Houston, TX, United States; ^4^Center for Precision Environmental Health, Baylor College of Medicine, Houston, TX, United States; ^5^Mitchell Center for Alzheimer's Disease and Related Brain Disorders, Department of Neurology, University of Texas Medical School at Houston, Houston, TX, United States

**Keywords:** epigenetic aging, RNAseq, induced pluripotent stem cells, postmortem brain, cocaine use disorder, opioid use disorder, dermal fibroblast cells, neurons

## Abstract

**Introduction:**

Human-derived induced pluripotent stem cell (iPSC) models of brain promise to advance our understanding of neurotoxic consequences of drug use. However, how well these models recapitulate the actual genomic landscape and cell function, as well as the drug-induced alterations, remains to be established. New *in vitro* models of drug exposure are needed to advance our understanding of how to protect or reverse molecular changes related to substance use disorders.

**Methods:**

We engineered a novel induced pluripotent stem cell-derived model of neural progenitor cells and neurons from cultured postmortem human skin fibroblasts, and directly compared these to isogenic brain tissue from the donor source. We assessed the maturity of the cell models across differentiation from stem cells to neurons using RNA cell type and maturity deconvolution analyses as well as DNA methylation epigenetic clocks trained on adult and fetal human tissue. As proof-of-concept of this model’s utility for substance use disorder studies, we compared morphine- and cocaine-treated neurons to gene expression signatures in postmortem Opioid Use Disorder (OUD) and Cocaine Use Disorder (CUD) brains, respectively.

**Results:**

Within each human subject (N = 2, 2 clones each), brain frontal cortex epigenetic age parallels that of skin fibroblasts and closely approximates the donor’s chronological age; stem cell induction from fibroblast cells effectively sets the epigenetic clock to an embryonic age; and differentiation of stem cells to neural progenitor cells and then to neurons progressively matures the cells *via* DNA methylation and RNA gene expression readouts. In neurons derived from an individual who died of opioid overdose, morphine treatment induced alterations in gene expression similar to those previously observed in OUD *ex-vivo* brain tissue, including differential expression of the immediate early gene EGR1, which is known to be dysregulated by opioid use.

**Discussion:**

In summary, we introduce an iPSC model generated from human postmortem fibroblasts that can be directly compared to corresponding isogenic brain tissue and can be used to model perturbagen exposure such as that seen in opioid use disorder. Future studies with this and other postmortem-derived brain cellular models, including cerebral organoids, can be an invaluable tool for understanding mechanisms of drug-induced brain alterations.

## 1. Introduction

As in many other fields, induced pluripotent stem cell (iPSC) (1) models have revolutionized studies in molecular psychiatry, delving deeper into human-specific molecular pathways than animal models (2–6). However, the degree of fidelity of these models to the personalized cellular environments and gene network interactions they aim to reproduce is unknown. Characterization of iPSC-derived lines by big transcriptomic and epigenomic data may better reflect the high dimensional complexity of human cell types than characterization by a few individual markers (3, 7). In particular, brain cellular maturity and aging can be measured by DNA methylation biomarkers known as epigenetic clocks (8). Since the link between DNA methylation and cellular aging was discovered, many clocks have been proposed that are trained on diverse tissue sets and reflect chronological and biological age or risk for aging-related disease (8–13). Moreover, several clocks have been specifically trained on embryonic and fetal tissue and stem cells, and used to characterize iPSC-derived cell lines (14–17).

Opioid use disorder (OUD) is a significant public health problem in the United States, with no FDA-approved options to facilitate abstinence, prevent relapse, or reduce damage caused by drug exposure. Although studies show substantial neurobiological abnormalities caused by OUD, including persistent alterations of gene expression (18–22), how opioids impact brain cell signaling networks is still not well understood, due in part to the difficulty in obtaining brain tissue from living individuals. Similar to OUD, cocaine use disorder (CUD) is a problematic pattern of drug use that puts individuals at risk for overdose, long-term health consequences (23), and lasting gene expression alterations in multiple brain regions (24–26), some of which overlap with OUD (27). While human postmortem brain tissue represents a highly valuable resource for studying substance use disorders such as OUD and CUD and is critical for examining the brain transcriptional landscape, it is difficult to control for human tissue covariates including sex and ancestry, integrity of RNA molecules and subsequent analysis quality is affected by postmortem interval (PMI), and any observations represent a static picture at the time of death (28–30). Additionally, it is not possible to discern whether the observed molecular profiles are due to chronic drug use, the untold number of environmental factors to which individuals were exposed during life, or the underlying genetic makeup.

The functional significance of postmortem brain findings can now be studied in humans at the cellular level using synthetic models derived from iPSCs. A great advantage of using iPSC-derived models is that the effect of outside environmental influences, such as infections or use of medications or multiple drugs, is removed, and only the genetic composition effects which are unchanged by transformation are left. iPSC-derived cellular brain models can therefore provide an important system for controlled pharmacological manipulation to elucidate drug-induced gene regulation mechanisms. Utilization of iPSCs derived from subjects with OUD as well as iPSC models of opioid exposure is currently limited (31). iPSC-derived dopaminergic neurons demonstrate differences in expression of dopaminergic system genes in subjects carrying a variable number tandem repeat polymorphism in DAT, and treatment with valproic acid altered dopaminergic gene expression specifically in cell lines from opioid addiction subjects (32). Another study used neuronal cell lines from subjects with a μ-opioid receptor (MOR) A118G single nucleotide polymorphism and identified neurophysiological and molecular differences that may predict altered opioid responsivity and/or dependence in this subset of individuals (33). A study investigating the effect of methadone on iPSC-derived cortical organoids found disruption of neural growth and suppression of neural electrochemical activity (34). iPSC derived neural spheroids have been used to screen for neuromodulatory activity and identify drug targets for opioid use disorder (35, 36). Other studies have investigated opioid roles in non-neuronal cell types including endothelial cells (37, 38) and cardiomyocytes (39). iPSC models have therefore proven to be a powerful yet underutilized tool for studying OUD and the effects of opioid drug exposure.

To further advance the use of iPSC-derived brain models in substance use disorders and examine the extent to which these models recapitulate the brain, in this study, we characterized a postmortem fibroblast-derived iPSC model of neuronal differentiation using DNA CpG methylation (DNAm) and RNA sequencing. Maturity of the model at different stages of stem cell induction and differentiation was assessed with immunofluorescent markers as well as by epigenetic aging and transcriptomic markers of cellular maturity. Different epigenetic clocks were assessed to optimally characterize both corresponding adult tissue and fibroblasts, as well as iPSCs and neurons from the same subjects. Further, using the postmortem-derived iPSCs, we created an *in vitro* neuron model of morphine and cocaine exposure in cell lines from individuals who died of an opioid or cocaine overdose and directly compared transcriptomic signatures of drug exposure in these lines to those observed in postmortem brain.

## 2. Methods

### 2.1. Acquisition of postmortem brain tissue and psychological autopsy from subjects

Dorsolateral prefrontal cortex (dlPFC) Brodmann Area 9 (BA9) brain tissue from Subject 1 (cocaine overdose death), Subject 2 (opioid overdose death), 7 additional cocaine use disorder (CUD) subjects and 13 controls absent of psychopathology was obtained from the University of Texas Health Science Center at Houston (UTHealth) Brain collection (UTHBC, RRID:SCR_022970) as previously described (19, 20). For each subject, demographics, autopsy and toxicology reports, and medical and psychiatric notes were collected. Consensus diagnoses of CUD according to DSM-5 criteria was determined by an independent panel of 3 clinicians based on review of all available records and a detailed psychological autopsy (40), where information regarding psychiatric clinical phenotypes (evidence of depression, mania, psychosis), drug use history, and any co-morbidities was obtained (Table 1; Supplementary Table 7). Importantly, all CUD subjects were exposed to only cocaine and not any other drug of abuse, including opioids and amphetamines.

**Table 1:** Postmortem subjects.

Subject	Sex	Age	Ethnicity/ancestry	Postmortem interval (hrs)	Cause of death	Postmortem psychiatric diagnosis
Subject 1	Male	58	Black	29.7	Toxic effects of cocaine	Cocaine Use Disorder
Subject 2	Female	36	White	24.4	Toxic effects of hydrocodone and morphine	Opioid Use Disorder, severe; Borderline Personality Disorder

### 2.2. Culture of postmortem fibroblasts and generation of induced pluripotent stem cells

Postmortem interval (PMI) was determined as the number of hours between time of death and time of autopsy. At autopsy, three 3 millimeter (mm) skin punches were collected from the forearm of Subject 1 and Subject 2 and dermal fibroblasts were cultured and expanded. Briefly, punches were dissected into 0.5 mm pieces in phosphate buffered saline (PBS) without calcium and magnesium (Corning, Corning, NY) with 100 micrograms (μg)/ milliliter (ml) primocin (InvivoGen, San Diego, CA) and 1x Pen/Strep (Gibco-Thermo Fisher Scientific, Waltham, MA), incubated in 2 units/ml type 2 collagenase (Worthington Biochemical, Lakewood, NJ) in Dulbecco’s Modified Eagle Media (DMEM, Corning) overnight, rinsed in DMEM and plated on 3 wells of a of a 24-well culture plate coated with Bovine plasma fibronectin (EMD Millipore, Darmstadt, Germany). Cells were cultured in fibroblast media consisting of 10% FBS (Gibco) and 1x PenStrep (Gibco) in DMEM and passaged and expanded using 0.05% Trypsin/0.53 millimolar (mm) EDTA (Corning) chemical dissociation. Cells were expanded into T-125 flasks and frozen to −80°C in a Mr. Frosty™ freezing container (ThermoFisher) at passage 3 in 1 ml of 10% dimethylsufoxide (DMSO, Sigma-Aldrich, St. Louis, MO) at 1.5 million cells per cryovial. Cells were transferred after 24 h (hr) to long term storage in liquid nitrogen until use when they were thawed quickly in a 37°C water bath, immediately diluted in fibroblast media, and centrifuged before plating to remove DMSO.

Postmortem fibroblasts were induced to iPSCs using the Cyto-Tune^™^-iPS 2.0 Sendai Reprogramming Kit (Invitrogen-Thermo Fisher Scientific, Waltham, MA), following manufacturer’s protocol. Briefly, fibroblasts were thawed and plated at passage 4 on 3 wells of a 6-well plates to 50% confluency and then were transfected overnight with the Cyto-Tune^™^-iPS 2.0 Sendai viral vectors in fibroblast media at the following Multiplicity of Infection (MOI): KOS = 5, hc-Myc = 5, hKlf4 = 3. After 6 days, cells were replated on 60 mm dishes coated with Geltrex^™^ LDEV-Free Reduced Growth Factor Basement Membrane Matrix (Gibco). Cells were maintained in TeSR^™^-E8^™^ media (StemCell Technologies, Vancouver, BC, Canada) and media was changed daily until iPSC colony formation (10–28 days). Colony clones were manually selected based on morphology and subsequently expanded in mTeSR^™^ Plus media (StemCell Technologies). Colonies were passaged for expansion using 10 micromolar (μM) EDTA in PBS without calcium and magnesium and gentle mechanical dissociation with a P1000 pipette. Two clones from each line were selected based on morphology and iPSC marker positivity for expansion and differentiation into neural cells. iPSCs between passage 7–13 were karyotypically analyzed by CellLine Genetics (Madison, WI) using standard G-banding technique.

### 2.3. Induction of neural progenitor cells and differentiation to neurons

Neural progenitor cells (NPC) were generated using StemCell Technologies Aggrewell^™^ plates to generate embryoid bodies (EBs) and then cultured using the StemDiff^™^ SMADi Neural Induction Kit, following manufacturer’s protocol as previously described (41). After 7 days, EBs were plated in Geltrex-coated 6-well plates in STEMDiff Neural Induction Medium + SMADi, and rosettes were manually selected 7 days later and replated on Geltrex-coated wells for expansion. NPCs were passaged at 80–90% confluence using Accutase cell detachment solution (Innovative Cell Tech, San Diego, CA) and expanded in Neural Basal (NB) media [50% DMEM/F12 (Corning), 50% Neurobasal Medium (Gibco), 1x GlutaMAX (Gibco), 1x NEAA (Gibco), 1x PenStrep (Gibco), 1x N-2 (Gibco), 1x B27 minus vitamin A (Gibco)] supplemented with 20 ng/ml of recombinant human FGF-Basic (1–155 a.a, PeproTech, Cranbury, NJ).

Cortical neurons were differentiated as previously described (41). NPCs were plated on Laminin/Poly-L-Ornithine coated 6-well plates at a density of 20,000 cells/cm^2^ and media was replaced the next day with Neuron Differentiation (ND) media consisting of NB media without FGF-Basic supplemented with 20 nanogram (ng)/ml BDNF (PeproTech), 20 ng/ml GDNF (PeproTech), 1 mm dibutyryl-cyclic AMP (Sigma-Aldrich), 200 nanomolar (nM) ascorbic acid (Sigma-Aldrich), 10 ng/ml IGF-1 (PeproTech), and 10 ng/ml WNT-3A (R&D Systems, Minneapolis, MN). ND media was changed every other day until the final neuron time points of Day 7, Day 14, or Day 21.

To avoid cell passage number affecting epigenetic age and cellular maturity, all cell samples were collected, induced and differentiated at the same passage number: passage 4 for fibroblasts, passage 16 for iPSCs, and passage 4 for NPCs. Neuronal differentiation time was based on only number of days cells spent in ND Media with neuronal growth factors: NPCs at Day 0, and neurons at Days 7, 14, and 21. Total differentiation time was approximated based on number of days cells underwent directed differentiation by NPC induction and neuronal differentiation, negating passaging and expansion of iPSCs and NPCs: iPSCs at Day 0, NPCs at Day 16, and D7, D14, and D21 neurons at Days 23, 30, and 37, respectively. For all DNA and RNA samples, cells were lifted in Trypsin, Accutase or 10 μM EDTA in PBS without calcium and magnesium, pelleted in 1.5 ml tubes and stored at -80°C until use. Fibroblasts were pelleted at 1.5 million cells, iPSCs were pelleted from 2 wells of a 6 well plate at 60–80% confluence, NPCs were pelleted from 2 wells of a 6 well plate at 100% confluence, and neurons were pelleted from 2 wells of each 6 well differentiation plate. In total, fibroblast lines from 2 postmortem subjects produced 4 total iPSC clones, and 4 clonal NPC and neuron lines.

### 2.4. Immunofluorescent staining

Immunocytochemistry was performed on iPSCs, NPCs, and D21 neurons mounted on Geltrex or Laminin/Poly-L-Ornithine coated coverslips. Coverslips were fixed in 4% paraformaldehyde in PBS and blocked for 2 h at room temperature in blocking buffer consisting of 2.5% goat serum, 2.5% donkey serum. Cells were incubated overnight at 4°C in primary antibody mixture in blocking buffer, washed 5 times for 5 min each, and then incubated in secondary antibody mixture in blocking buffer for 1 h at room temperature. Supplementary Table 1 lists primary and secondary antibody concentrations for each experiment. Nuclei were counterstained with DAPI, washed 3 times, and mounted on slides for confocal microscopy.

### 2.5. Neuron drug treatment

10 mM morphine stocks were prepared by dissolving morphine sulfate (Sigma-Aldrich, M8777) in PBS. 10 mM cocaine stocks were prepared by dissolving cocaine hydrochloride (Sigma-Aldrich C5776) in PBS. Stocks of cocaine and morphine were stored at 4°C until use at which time either morphine or cocaine was diluted in NDM to produce a final concentration of 10 µM. Acute neurotoxicity in morphine-treated NPC and D21 Neuron cultures was determined by Roche (Indianapolis, IN) Cell Death Detection ELISA kit following manufacturer’s protocol. 10 µM doses of morphine and cocaine were chosen for treatment as they caused significant changes in gene expression yet did not cause acute neurotoxicity. Acute neurotoxicity in cocaine-treated NPC and D21 neuron cultures was determined by assessing cell viability using Abcam (Cambridge, United Kingdom) Cell Counting Kit 8 following manufacturer’s protocol. To model chronic drug exposure in OUD and CUD, neuron lines derived from two iPSC clones from one individual with OUD who died of an opioid overdose (morphine and hydrocodone, Table 1, Subject 2) were treated with 10 µM morphine or left untreated for 7 days during differentiation from day 14 to day 21 of the neuron differentiated protocol. Morphine-containing or morphine-free media was changed daily for 7 days. Neuron lines derived from two iPSC clones from one individual with CUD who died of a cocaine overdose (Table 1, Subject 1) were treated with 10 µM cocaine or left untreated for 7 days during differentiation from day 14 to day 21 of the neuron differentiated protocol. Cocaine-containing or cocaine-free media was changed daily for 7 days. For each cell line, treated and untreated cells were pelleted at day 21 of differentiation for RNA seq analysis.

### 2.6. Next-generation RNA sequencing, genotyping microarray and differential expression analysis

RNA extraction, library preparation and RNAseq analysis was performed on flash frozen postmortem BA9 Brain Tissue samples as previously described (20). For cell samples, RNA was collected from dry frozen pellets using the RNeasy Plus Mini kits (Qiagen, Hilden, Germany). 400 ng RNA per sample was used for mRNA library construction using NEBNext^®^ Ultra^™^ RNA Library Prep Kit (Illumina Inc., San Diego, CA). Paired-end sequencing reads (150 bp) were generated on an Illumina HiSeq2000 platform (Novogene Bioinformatics Institute, Chula Vista, CA).

Sample sequencing quality was assessed using FastQC (Babraham Bioinformatics, Cambridge, United Kingdom). Reads were mapped to the GRCh38 genome using STAR version 1.7.10a (42). Gene and transcript counts were obtained using featureCounts (43), and from the raw counts, gene lengths, library size, and counts per million were calculated. Genes with CPM > 1 in at least 1 sample were kept for analysis.

All statistics were performed using R statistical computing programming language unless otherwise noted (44). RNA cell type and maturity deconvolution was performed as previously described using minfi package projectCellType() function (7, 41, 45). Supplementary Tables 3B,C contains the deconvolution design matrices employed for analysis.

To calculate principal components (PC) to account for genetic background and ancestry of human subjects in downstream differential expression analyses of CUD subjects and controls, genome-wide single nucleotide polymorphism (SNP) genotyping was performed on blood from postmortem subjects using the Infinium Global Screening Array(GSA)-24 kit (version 3.0, Illumina, San Diego, CA) according to manufacturer’s instructions. Infinium GSA chips were read on the illumina iScan and exported as raw intensity data (.idat) files. Raw.idat files were exported to plink1.9 (46) .ped format using GenomeStudio using GRCh38 as reference genome. SNPs were filtered using the following parameters in plink: allele frequency > 0.01, Hardy–Weinberg equilibrium exact p-val < 1e-6, missing call rate < 0.1. SNP strands were flipped to reference chromosome using SAMtools bcftools (47, 48). Preprocessed and filtered SNPs were uploaded to the Michigan TOPmed server (49) for imputation, using the NHLBI TOPmed reference panel (50) and minimac2 (51). Imputed SNPs were again filtered using the following parameters in plink: allele frequency > 0.01, Hardy–Weinberg equilibrium exact p-val < 1e-6, missing call rate < 0.1. SNP strands were again flipped to reference chromosome and mismatched and duplicated SNPs were removed. Principal component analysis was performed on filtered SNPs using plink and the first 6 PCs were extracted to be used as covariates in differential expression analysis. Other human tissue covariates of RNA integrity number (RIN), postmortem interval (PMI), and cerebellar pH were calculated as previously described (20). Means of continuous covariates (Age, pH and RIN) were compared between CUD and controls using Student’s t-test after validating conformity to normal distribution. Enrichment for binary variables (sex, black ancestry) in CUD vs. control was determined by Fisher’s exact test.

Differential expression (DE) analysis was performed on mRNA of drug-treated cell lines compared to untreated cells, and postmortem brain of CUD subjects and controls using R Bioconductor package EdgeR with upper quartile normalization (52, 53). For drug-treated cell lines, pairwise comparisons between clones were made using the formula (~Clone + Treatment). For postmortem brain CUD analysis variance partition analysis and canonical correlation analysis on patient demographics using R package variancePartition (54) revealed the covariates sex, age, PMI, RIN, PH, and Geno PCs 1–6 accounted for a sufficient proportion of variance not due to disease (Supplementary Figure 3), and all of these were accounted for in the differential expression model. PC analysis of brain transcriptomes was performed using R stats prcomp function. Differentially expressed genes (DEGs) were determined using the following cutoffs: absolute fold change (|FC|) > 1.5, Benjamini-Hochberg adjusted value of p with false discovery rate (FDR) <0.2 or value of *p* <0.05. For comparisons with OUD, DE genes and normalized (CPM) counts from dlPFC (BA9) in OUD and corresponding controls were extracted from previously published data [(20); GEO series accession number GSE182321],^1^ with significance cutoffs as nominal value of *p* <0.05 and |FC| > 1.5 for the purposes of integration with iPSC-neuron data. The control subjects used in Mendez et al. (20) were the same ones compared to CUD subjects in this study.

Gene Ontology analysis was performed on nominally significant (|FC| > 1.5 and value of p <0.05) genes in brain and cocaine or morphine-treated cells using TopGO (55) to query GO annotation in 2 categories: biological processes (BP) and molecular function (MF). Classic weight correction algorithm was employed and significant GO terms were determined by Fisher’s exact tests.

### 2.7. DNA methylation microarray and epigenetic aging analysis

DNA extraction and DNA methylation microarray was performed on flash frozen postmortem BA9 brain samples as previously described (19). For cell samples, DNA from frozen pellets was isolated using the DNeasy Blood & Tissue Kit (Qiagen). 500 ng of DNA were bisulfite-converted with the EZ DNA Methylation Kit (Zymo Research, Irvine, CA) and hybridized for the Infinium MethylationEPIC kit (Illumina). Sample chips were read on the iScan microarray reader (Illumina), according to the manufacturer’s instructions, generating raw IDAT file for downstream analyses.

CpG detection value of ps and beta values were calculated for cells and brain tissue using the minfi R package (45) and annotated to GRCh37 (hg19). Probes with detection value of *p* <0.05 were kept for analysis. Horvath Multi Tissue Clock age scores and GrimAge scores were calculated using the online DNAmage calculator.^2^ Steg Fetal Clock and Knight Gestational Age Clock scores were calculated as previously described (15, 17). In order to account for the paired nature of our experimental design and aging of cell types accounting for subject and clone origin, predictive quality of each epigenetic clock was determined by fitting Bayesian linear mixed effects models with cell line and clone as random effects. The model was fit to a wishart prior with a random slope and intercept [Total_Differentiation_Time ~ EpiAge + (1 + EpiAge| CellLine)] using Bayesian linear effects model (blme) R package (56).

## 3. Results

### 3.1. Generation and validation of postmortem fibroblast iPSC model

**Figure 1:**
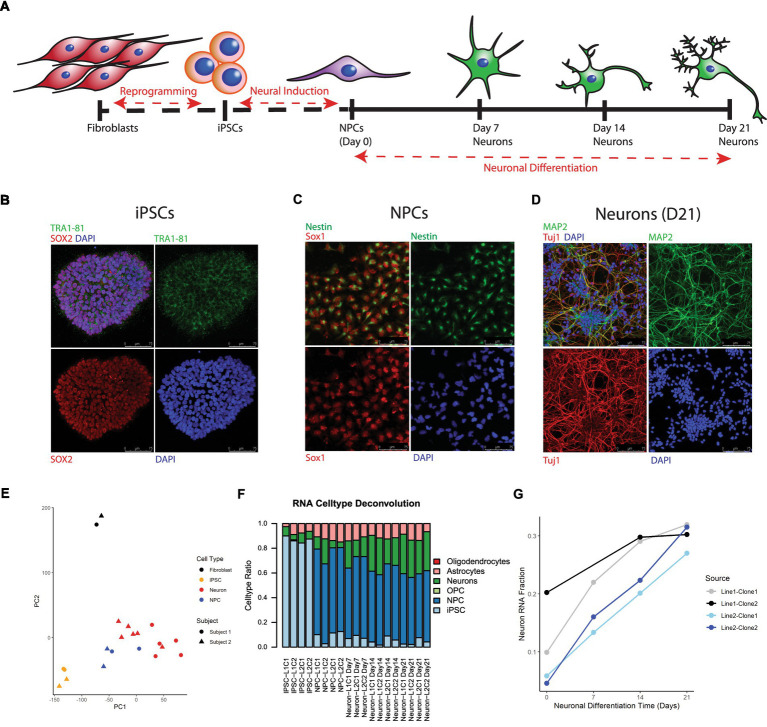
Differentiation and transcriptional characterization of cell lines. **(A)** Scheme of postmortem fibroblast reprogramming to iPSCs and differentiation to neurons. **(B)** Immunofluorescent (IF) staining of iPSC colonies for the stem cell markers TRA 1–81 (green), SOX2 (red), and DAPI (blue). **(C)** IF staining of NPCs for the NPC markers Nestin (green) and Sox1 (red). **(D)** IF staining of Day 21 neurons for the neuronal markers Map2 (green), Tuj1 (red) and DAPI (blue). **(E)** Principal Component Analysis (PCA) of RNA seq data, colors label cell type and shape label postmortem subject source. **(F)** Stacked barplot showing results of RNA cell type deconvolution. **(G)** Neuron RNA cell type fraction plotted against neuronal differentiation time, NPCs noted as time = 0. Color of plot line indicates cell line and clone source.

iPSCs were successfully generated from cultured postmortem human fibroblasts and subsequently differentiated into 21-day-old neurons (Figure 1). As stated above, these cells were sourced postmortem from 2 subjects (Lines 1 and 2), and 2 iPSC clones were chosen from each induced fibroblast cell line, yielding a total of 4 neuronal cell lines: L1C1, L1C2, L2C1, and L2C2 (Table 1). All iPSC lines were karyotypically normal (data not shown). RNA and DNA was collected for next generation RNA sequencing and methylation analysis, at 6 different time points of induction and differentiation for each cell line: cultured postmortem fibroblasts, iPSCs, NPCs, and Day 7 (D7), Day 14 (D14), and Day 21 (D21) Neurons (Figure 1A). One sample, Neuron Line 1 Clone 2 (L1C2) Day7, was excluded from RNA seq analysis due to poor RNA yield. Immunofluorescence-stained iPSCs, NPCs, and D21 Neurons showed a majority of cells were positive for markers of iPSCs (TRA1-81, and SOX2), NPCs (Nestin and Sox1), and Neurons (MAP2, Tuj1), respectively (Figures 1B–D).

Principal component analysis (PCA) of RNA seq data revealed that cell transcripts visually cluster by cell type along PCs1 and 2 (Figure 1E). Cell type deconvolution of RNAseq data showed that the NPC and Neuron proportion of cell transcripts increased while the iPSC proportion decreased during differentiation from iPSCs to Neurons (Figure 1F; Supplementary Table 3), and that specifically the Neuronal RNA fraction increased progressively over neuron differentiation (Figure 1G). Neuron RNA fraction correlated strongly with days of neuronal differentiation (Pearson correlation coefficient = 0.88, 95%CI = [0.65,0.96], *p* value <0.00001).

### 3.2. Postmortem frontal cortex epigenetic age parallels that of cultured skin fibroblasts and chronological age

**Figure 2:**
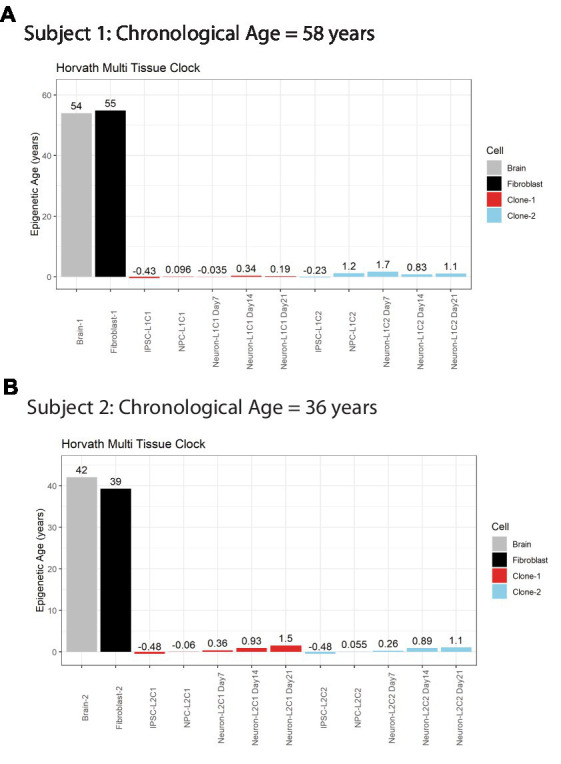
Horvath Adult epigenetic age of human brain vs. fibroblasts. **(A)** Horvath age scores for Subject #1 **(A)** and Subject #2 **(B)**, labeled by cell line or tissue. Colors indicate cell source, age scores above bars.

A major advantage of postmortem-derived fibroblast cell lines is that the cultured cells and subsequent iPSCs-derived neural lines can be directly compared to the same subject’s brain. To assess how postmortem fibroblast culture and iPSC induction and differentiation compared with the epigenetic age of the corresponding isogenic brain tissue, the Horvath Multi Tissue Clock epigenetic age (10) was calculated based on DNA methylation signatures from brain tissue and cell lines (Figure 2). This clock notably correlates strongly with chronological age. In both postmortem subjects, the calculated Horvath age of prefrontal cortex tissue and cultured fibroblasts were near the person’s chronological age at time of death (Figure 2).

### 3.3. Stem cell induction from fibroblasts resets epigenetic clock

iPSC transformation of fibroblasts effectively reversed the Horvath Multi Tissue Clock epigenetic age of the cells to zero, and in some cases to a negative age (Figure 2). Importantly, all cells differentiated from these iPSCs remained very young throughout differentiation. Differentiation of the cells to NPCs and then neurons tended to increase the Horvath age to a more positive score. However, this increase was small, less than 2 years at all time points, and not all cell lines aged progressively in this way (Figure 2A).

### 3.4. Steg fetal brain clock predicts cellular maturity more effectively than adult clocks

**Figure 3:**
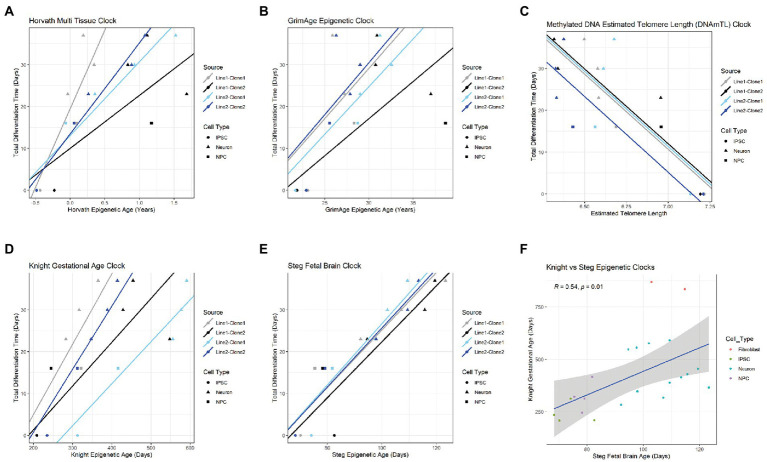
Comparison of multiple epigenetic clocks *via* Bayesian linear mixed model. Plots comparing Horvath Adult Epigenetic Clock **(A)**, GrimAge Clock **(B)**, DNAmTL Clock **(C)**, Knight Gestational Age Clock **(D)** and Steg Fetal Brain Clock **(E)**. For each plot, age score is plotted against approximate total differentiation time (from iPSC stage) and linear effects model for each cell clone is demarcated by the plot line. Colors indicate clone, shapes indicate cell type. **(F)** Correlation plot between Knight Gestational Age Clock and Fetal Brain Clock, Pearson correlation coefficient and value of p labeled at top. Cell type noted by color. Linear regression line labeled surrounded by gray standard error field.

Epigenetic age scores were also calculated for other standardly used epigenetic clocks: the GrimAge clock (12), DNAm estimated Telomere Length (DNAmTL) (13), the Steg Fetal Brain Clock (17), and the Knight Gestational Age Clock (15) (Supplementary Figure 2). To determine which clock(s) best predicts cell differentiation state, Bayesian linear mixed effects models were fitted to approximate total days of differentiation within each cell line for the Horvath Multi Tissue Clock and each other additional clock (Figures 3A–D; Supplementary Table 2). Statistics for each model is shown in Table 2.

**Table 2:** Bayesian linear mixed effects model statistics for epigenetic clocks predicting total differentiation time.

Epigenetic clock	Coefficient	Std. error	*t* statistic	*p* value
Horvath (Years)	22.46	9.17	2.45	0.014
Steg (Days)	0.7	0.25	2.78	0.0054
aKnight (Days)	0.13	0.44	0.29	0.77
GrimAge (Years)	2.24	0.84	2.67	0.0075
DNAmTL	−35.98	5.41	−6.65	2.93E-11

Of all models tested, the Steg Fetal Brain Clock best predicted days of differentiation per line (standard error = 0.25, *p* = 0.0054; Figure 3E). However, the Horvath Multi Tissue Clock and GrimAge Clock also correlated fairly well with the cell differentiation timeline (Figures 3A,B; Table 2). The GrimAge Clock did not revert the iPSC and neural cell line ages to near zero as the Horvath clock did, predicting cell age ranges from 20 to 40 years (Supplementary Figures 1A,E). The DNAmTL Clock converged on a model with a strong effect coefficient (coefficient = −35.98, *p* = 2.93e-11), yet the scores fit the differentiation time points relatively poorly (standard error = 5.41).

**Figure 4:**
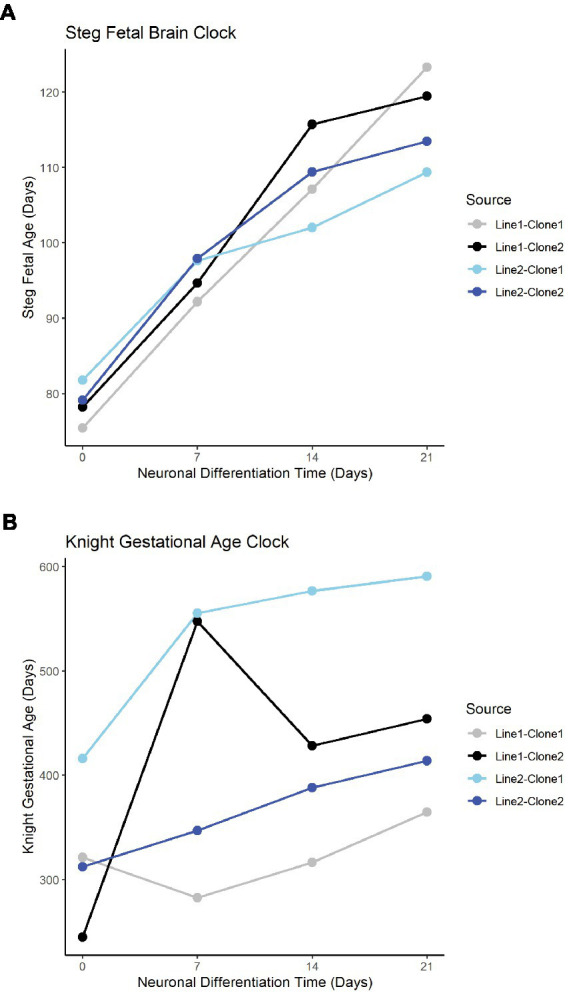
Fetal clocks plotted against neuronal differentiation time for Steg Fetal Brain Clock **(A)**, and Knight Gestational Age Clock **(B)**. NPCs noted as time = 0. Color of plot line indicates cell line and clone source.

The Steg Fetal Brain Clock and Knight Gestational Age Clock scores correlated significantly with each other, but the Steg clock provided superior discrimination between major cell types (Figure 3F). The Steg Fetal Brain Clock was also superior to the Knight Gestational Age Clock following neuronal differentiation from neural progenitor cells (Figure 4).

### 3.5. Steg fetal epigenetic clock correlates closely with transcriptional cellular maturity

**Figure 5:**
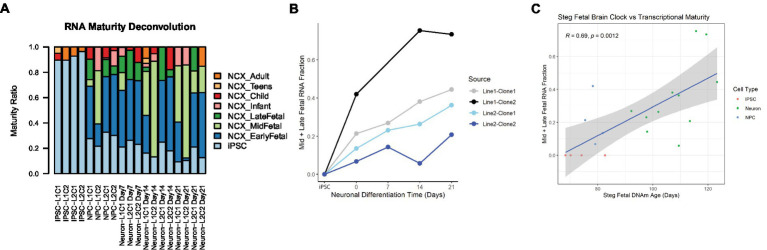
RNA transcriptional maturity across differentiation. **(A)** Stacked barplot showing results of RNA maturity deconvolution. **(B)** RNA maturity score indicated by combined Mid and Late fetal fraction plotted against neuronal differentiation time, NPCs noted as time = 0 Days, iPSCs labeled. Color of plot line indicates cell line and clone source. **(C)** Correlation plot between Steg Fetal Brain Clock and RNA Mid + Late Fetal transcriptional maturity, Pearson correlation coefficient and value of p labeled at top. Cell type noted by color. Linear regression line labeled surrounded by gray standard error field.

In addition to cell type, RNA mixtures can also be used to deconvolve sample maturity (7), and this transcriptional maturity estimation was applied to the postmortem iPSC lines and neurons (Supplementary Table 3). While the largest proportion of cell transcripts remained at iPSC or fetal maturity throughout the differentiation process, as cells differentiated from iPSCs to NPCs and then to Neurons, the RNA fraction corresponding to immature iPSCs decreased, while the Mid to Late Fetal RNA fraction increased (Figure 5A). Using the Mid and Late Fetal RNA fractions as a measure of later transcriptional maturity, the RNA maturity of the differentiating neurons increased per line by days of differentiation (Figure 5B). Finally, the Steg Fetal Brain Clock was correlated strongly with the Mid and Late Fetal RNA maturity fraction (Figure 5C, Pearson correlation coefficient = 0.69, 95%CI = [0.34,0.87], *p* < 0.001), integrating two models of cellular aging and maturity.

### 3.6. Morphine-treated cells recapitulate gene expression signatures of isogenic postmortem opioid overdose brain

**Figure 6:**
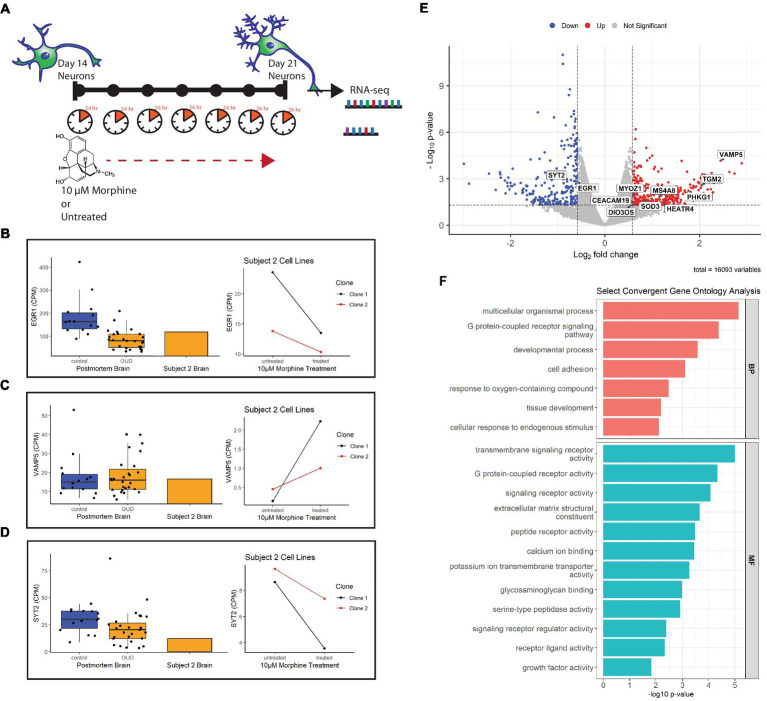
Gene expression of morphine-treated cell lines. **(A)** Morphine treatment paradigm of Subject 2 neuron cell lines. Cells were treated with 10 μM morphine or left untreated for 7 days, between day 14 and 21 of differentiation. RNA was collected from treated and untreated cells at 21 days of differentiation. **(B)** Volcano plot showing differentially expressed genes in the morphine-treated cell model. Genes convergent with those in postmortem brain of OUD subjects are labeled. For data integration with postmortem brain OUD gene signatures, a nominal value of p cutoff of 0.05 and absolute FC of at least 1.5 (depicted as log2FC = ± 0.58 in volcano plot) was used to determine significance. **(C–E)** Boxplots (left) of postmortem brain gene expression (CPM) compared with barplot (middle) of postmortem brain from Subject 2. Line plots (right) show gene expression changes per neuron clonal cell line after morphine treatment. **(F)** Selected convergent biological processes (BP) and molecular functions (MF) perturbed in both postmortem brain of OUD subjects and morphine-treated cell lines.

Previous studies in our lab found differential expression of several neuron-related genes and pathways in the dorsolateral prefrontal cortex (dlPFC) of individuals with opioid use disorder (20). To determine whether these gene expression signatures can be effectively modeled in postmortem-derived neurons, a 7 day drug treatment experiment as a proxy for chronic opioid exposure was designed as proof-of-concept to test parallels between opioid-exposed iPSC-derived neurons from Subject 2 [who died of a hydrocodone and morphine overdose and suffered from OUD during life (Table 1)] and the alterations identified in opioid-exposed postmortem brain tissue. Importantly, the iPSC-derived neurons express genes for the mu, kappa and delta opioid receptors: OPRM1, OPRK1, OPRD1 (Supplementary Figure 2). Figure 6A describes the chronic morphine treatment paradigm during neuron differentiation. As a measure of acute neurotoxicity, no significant cell death of NPCs or neurons was observed after a single 24 h 10 μM morphine treatment (data not shown).

Using FDR < 0.2 and |FC| > 1.5 as cutoffs, we identified 166 upregulated and 146 downregulated genes after morphine treatment in neurons (Supplementary Table 4, bold). For downstream data integration with differentially expressed genes in OUD postmortem brain (20), significance criteria were expanded to include nominal value of *p* <0.05 and |FC| > 1.5, which found 340 upregulated and 236 downregulated genes (Figure 6B). From this, 20 genes were found dysregulated in both OUD dlPFC and morphine-treated neurons, with 11 of these genes, including EGR1, VAMP5, and SYT2 being concordantly dysregulated (e.g., concurrently up or down in both brains and cells; Figure 6; Supplementary Table 5). For each of the concordant genes, Subject 2’s postmortem dlPFC expression reflected the up- or downregulation of the entire OUD brain cohort, and treatment of the postmortem-derived neurons with morphine recapitulated the effect of opioid exposure. Additionally, gene ontology analysis revealed several key biological pathways and molecular functions similarly perturbed in both the morphine-treated neurons and brains from OUD subjects, including G protein-coupled receptor activity, extracellular matrix structure, calcium ion binding, cell adhesion, tissue development, and growth factor activity (Supplementary Table 6; Figure 6F).

### 3.7. Cocaine-treated cells share gene expression signatures of postmortem brain from cocaine-dependent subjects

Eight postmortem CUD subjects and 13 controls were chosen from the UTHealth Brain Collection for differential expression analysis (Supplementary Table 7). CUD subjects were more likely to be black vs. white than controls (Fisher exact test value of *p* = 0.049), and had a higher pH (Student’s *t*-test value of *p* = 0.024). There was no statistically significant difference between the groups in sex, age, PMI or RIN. Canonical correlation analysis revealed that a phenotype of CUD correlated strongly with both ancestry (R = 0.533) and pH (0.475; Supplementary Figure 3A). Variance partition analysis revealed that ancestry contributed to the most variance in the data (Supplementary Figure 3B), and that the first 6 calculated genotype principal components (GenoPC1-6) all contributed significantly to gene expression variance (Supplementary Figure 3C). After adjusting for sex, age, PMI, RIN, pH, and GenoPC1-6, variance contribution of all covariates, including ancestry was substantially reduced (Supplementary Figure 3D). CUD and control subjects did not visually cluster on the first 2 principal components (Supplementary Figure 4A). Using nominal value of *p* < 0.5 and |FC| > 1.5 as cutoffs, 91 genes were downregulated and 87 genes upregulated (Supplementary Figure 4B; Table 8). Of these, only 2 genes, LIN28A and C3AR1, withstood FDR correction <0.2.

To explore how well cocaine-exposed neurons could model the postmortem brain gene expression effects of cocaine, we compared gene expression signatures of neurons from Subject 1 (who died of cocaine overdose and suffered from cocaine use disorder during life) to those genes differentially expressed between CUD and control in BA9. Supplementary Figure 4C describes the chronic cocaine treatment paradigm during neuron differentiation. As a measure of acute neurotoxicity, no significant reduction of cell viability in NPCs or neurons was observed after a single 24 h 10 μM cocaine treatment (data not shown). Using FDR < 0.2 and |FC| > 1.5 as cutoffs, we identified 166 upregulated and 146 downregulated genes after cocaine treatment in neurons (Supplementary Figure 4D; Table 9, bold). Three genes were significantly dysregulated in both CUD BA9 and cocaine-treated neurons, LIN28A, POPDC2, and PRR29 (Supplementary Figures 4D–F; Table 10) but only LIN28A was concordantly downregulated in both cocaine-exposed brain and cells. Convergent gene ontology analysis revealed that both cocaine-exposed brain and cells had differential biological pathways enriched for signal transduction, cell communication, and response to stimulus, as well as differential molecular functions in transmembrane signaling receptor activity, GPCR activity, and extracellular matrix structure (Supplementary Figure 4G; Table 11).

## 4. Discussion

An important promise of human-derived synthetic neural cell models is that these could mimic the human brain better than animal models and serve as a more robust experimental system to test hypotheses about pathophysiology, treatment targets, and treatment response. However, to achieve this, a critical first step is to determine to what extent the genomic landscape and the cellular interactions of the cell models recapitulate that of actual brain tissue. This study offers the unprecedented opportunity to examine the extent to which iPSC-derived neuronal models recapitulate the brain from the same subject from which they were derived, thereby addressing the limitations in studies using either *ex-vivo* human brain or synthetic cell models individually. In summary, we have introduced a novel iPSC-derived neuronal differentiation system from postmortem fibroblasts. This *in vitro* model was characterized using several different readouts, including immunofluorescent staining for key markers, cellular transcriptional maturity using RNA seq deconvolution, and epigenetic maturity from several adult and fetal epigenetic clocks. Finally, as a proof-of-concept study, opioid and cocaine-induced alterations in iPSC neurons were directly compared to those observed in *ex-vivo* OUD or CUD, respectively, and to the source subjects’ brain tissue.

The epigenetic age of *ex-vivo* brain tissue paralleled the chronological age of the deceased persons as well as the epigenetic age of their isogenic cultured postmortem fibroblasts. Cellular aging is a critical process during neurodevelopment and maturation (17). Cellular aging can be measured by DNA methylation biomarkers known as epigenetic clocks (8). Several clocks have been specifically trained on embryonic and fetal tissue and stem cells and have been used to characterize cellular maturity during neuronal differentiation (14–17).

When assessing epigenetic aging and cellular maturity, it may be prudent to use multiple clocks for different purposes. For instance, in our study, the Horvath Multi Tissue Clock that correlates with chronological age was useful to compare each subjects’ chronological age at time of death with the biological epigenetic aging of the brain and the cultured fibroblast lines. Clocks trained on fetal and embryonic tissue are important tools for assessing maturity of stem-cell derived cell models. We found the Steg Fetal Brain Clock was superior for discriminating neuronal differentiation stages in the iPSC-derived neurons, although all clocks assessed correlated with cellular differentiation time.

Induction of stem cells from adult postmortem fibroblasts essentially zeroed out the Horvath Multi Tissue Clock, as expected based on previous studies (10, 14). Interestingly, this was not the case for the GrimAge clock, an adult epigenetic clock that predicts lifespan and morbidity and mortality by DNA methylation that correlates with plasma biomarkers of diseases of aging (12). While this could mean that these CpG sites contributing to age-related morbidity and mortality are maintained after stem cell induction and differentiation, and could be probed for aging studies, further studies including a larger paired biological sample size are necessary to determine if this is the case. The DNAmTL clock converged on a significant linear model, but fit the data with a high standard error. This could be due to a nonlinear effect of differentiation causing cell cycle arrest as the NPCs differentiate into neurons and subsequently cease telomere length attrition, as has been previously reported in brain neurons (57).

iPSC neurons are notably transcriptionally immature, corresponding to human embryonic or fetal developmental stage (7), and this was seen in our postmortem fibroblast-derived iPSC neurons. As the neurons differentiated, the proportion of transcripts corresponding to later fetal neocortex stages increased. Therefore, mid to late fetal proportion proved to be a useful transcriptional readout score for neuronal maturity that could be compared with the cells’ epigenetic data. RNA transcriptional maturity and DNAm Steg epigenetic aging scores were strongly correlated as cells were differentiated, indicating their value as two robust molecular models of cellular maturity in iPSC neurons.

One downside of modeling diseases using iPSC models is that the dedifferentiation process to induce the stem cells effectively erases many of the disease, aging, and environmental epigenetic signatures from the source patient, a phenomenon known as epigenetic rejuvenation (6, 58, 59). However, this epigenetic reprograming can be useful in designing experiments to answer questions related to an individual perturbagen, such as consequences of drug exposure in substance use disorders or drug overdose (31). It is important to note that OUD and CUD are an incredibly complex diseases characterized by behavioral drug seeking, tolerance, withdrawal, and psychosocial distress (60), and many aspects of the diseases are impossible to model in a dish with current technologies. Knowing this, we sought to use the postmortem-derived iPSC neurons to explore which transcriptional consequences of repeated drug exposure specifically recapitulate those seen in brain as a model of chronic drug use. To assess this, gene expression profiles from two morphine-treated clonal cell lines from an opioid overdose victim were compared to the same subjects’ brain and to a larger OUD postmortem cohort. Eleven differentially expressed genes in the morphine-exposed cells paralleled dysregulation directionality of OUD brains, including the immediate early gene EGR1, previously implicated in substance use disorders (61), and the SNARE protein genes VAMP5 and SYT2. The latter findings are of particular interest, as mu and delta opioid receptors directly inhibit the SNARE complex to contribute to synaptic remodeling (62). Importantly, the gene pathways enriched following morphine treatment in neurons paralleled gene expression signatures of OUD in postmortem brain. These included G-protein-coupled receptor (GPCR) pathways, of interest as the opioid receptors themselves are GPCRs; and developmental processes, which highlight the potential utility of iPSC-derived neurons as prime models for development studies.

To further establish our proof-of-concept model, gene expression profiles from two cocaine-treated clonal cell lines from a cocaine overdose victim were compared to a differential expression analysis of CUD subjects and controls. While the effect of cocaine treatment on the neuronal cell lines was fairly robust, power of the postmortem brain differential expression analysis of CUD versus controls was limited due to the small sample size and human tissue covariate interactions. Importantly, subjects selected for DE analysis were exposed to only cocaine and not any other drug of abuse, including opioids and amphetamines. While this selectivity is important for determining cocaine-specific effects, it limited sample availability. However, the preliminary analyses demonstrate overlap of gene signatures, in cocaine exposed brain and iPSC-neurons, particularly related to signal transduction, cell communication, and response to stimulus. Future studies using this model could be a powerful tool to disentangle the effects of individual drugs as well as drug interactions such as those between cocaine and morphine in polysubstance users.

Limitations to this study include a small sample size, as fibroblast cell lines were only isolated from two subjects due to labor intensive protocols. This is therefore considered a proof-of-concept study and future studies will include iPSC lines from more postmortem subjects as well as additional clonal replicates for each subject. However, because of the extensive characterization of clonal replicates reported here, we are confident that the postmortem cell model feasibly compares to other iPSC-derived neuron cell lines, with the added value of the ability to compare terminally differentiated neurons to isogenic brain. Future studies with lines generated from more subjects could validate the opioid and cocaine drug exposure effects observed here and could also be used to investigate effects of other perturbagens such amphetamines, alcohol or even therapeutics. Further work is also necessary to ascertain how much epigenetic and transcriptomic variability is due to biological variables between samples versus technical differences in iPSC line generation and differentiation. For example, while all neuron lines expressed mu, kappa, and delta opioid receptor genes, Subject 2 clone 2 mu opioid receptor expression was 26 times higher than clone 1, which could contribute to variability in downstream gene pathways. Additionally, as stated above, the pluripotency induction process clears many disease- and aging-related epigenetic signatures that are acquired during life, as was seen in our data. Other cell modeling technologies, such as direct lineage reprogramming into neurons (63) maintain many of the epigenetic signatures from the source tissue, including those related to cell aging, and may be useful to explore the epigenetic effects of human exposures that cannot be adequately modeled in a dish (64). Another limitation is the focus on one drug concentration and time of exposure. While the 10 µM drug treatment paradigm was geared to produce chronic effects as a proxy to repeated exposure seen in OUD or CUD, this may not accurately reflect the exposure of many chronic users. Future studies with variable concentrations and time courses will be useful to compare with isogenic brains that are exposed to different drugs to determine acute versus chronic effects. Finally, our current study focused on one brain cell type, neurons, which we compared to bulk brain tissue composed of heterogenous brain cell types. In the future, studies differentiating postmortem-derived iPSCs into cerebral organoids (65) could better approximate whole brain tissue signatures (66, 67), while single-cell RNAseq of the brain tissue could elucidate more robust cell type-specific signatures.

In conclusion, this novel neuronal model derived from postmortem human fibroblasts promises to be a powerful tool to investigate genetic diseases, drug/toxin exposure, and cell maturation during development, and allows comparison with isogenic brain tissue. Future studies utilizing this model to explore cellular and molecular facets of brain physiology and disease will be invaluable in the field.

## Data availability statement

The original contributions presented in the study are publicly available. The RNA-Seq data used in this study from postmortem Brodmann Area 9 of opioid use disorder subjects and controls was previously published (20) and can be found at NCBI GEO (GSE182321, https://www.ncbi.nlm.nih.gov/geo/query/acc.cgi?acc=GSE182321). New RNA-Seq data generated for this study from the cell lines used and postmortem Brodmann Area 9 of cocaine use disorder subjects has been deposited at NCBI GEO (GSE224096; https://www.ncbi.nlm.nih.gov/geo/query/acc.cgi?acc=GSE224096).

## Ethics statement

The studies involving human participants were reviewed and approved by University of Texas Health Science Center at Houston Institutional Review Board. Written informed consent for participation was not required for this study in accordance with the national legislation and the institutional requirements. IRB approval to contact next of kin for brain and skin tissue donation, and for performance of psychological interviews regarding deceased individuals is approved *via* protocol HSC-MS-15-0247—The UTHealth Brain Collection for Research in Psychiatric Disorders PI: Consuelo Walss-Bass, UTHSCH.

## Author contributions

CW-B and EM: conceptualization and visualization. EM, SG, GF, and CC: methodology, software, and formal analysis. EM, LS, DG, SM, KN, KM, and TM: investigation and data curation. GF, CC, and CW-B: resources, supervision, and funding acquisition. EM, SG, DG, GF, CC, and CW-B: writing-original draft. All authors contributed to the article and approved the submitted version.

## Funding

CW-B was partially supported by grant R01DA044859. SG and CC were partially supported by The Cancer Prevention Institute of Texas (CPRIT) grants RP170005, RP210227, RP200504, and NIH P30 shared resource grant CA125123, NIEHS grants P30 ES030285, and P42 ES027725, and NIMHD grant P50 MD015496. GF was partially supported by the Baszucki Brain Research Fund.

## Conflict of interest

The authors declare that the research was conducted in the absence of any commercial or financial relationships that could be construed as a potential conflict of interest.

## Publisher’s note

All claims expressed in this article are solely those of the authors and do not necessarily represent those of their affiliated organizations, or those of the publisher, the editors and the reviewers. Any product that may be evaluated in this article, or claim that may be made by its manufacturer, is not guaranteed or endorsed by the publisher.

## Footnotes

^1^ https://www.ncbi.nlm.nih.gov/geo/query/acc.cgi?acc=GSE182321

^2^ https://dnamage.genetics.ucla.edu/new
