# Supplementary figures and images for: A human stem cell-derived neuronal model of morphine exposure reflects brain dysregulation in opioid use disorder: Transcriptomic and epigenetic characterization of postmortem-derived iPSC neurons
Source: Front Psychiatry. 2023 Feb 16;14:1070556. doi: 10.3389/fpsyt.2023.1070556 (PMC9978009; doi:10.3389/fpsyt.2023.1070556)

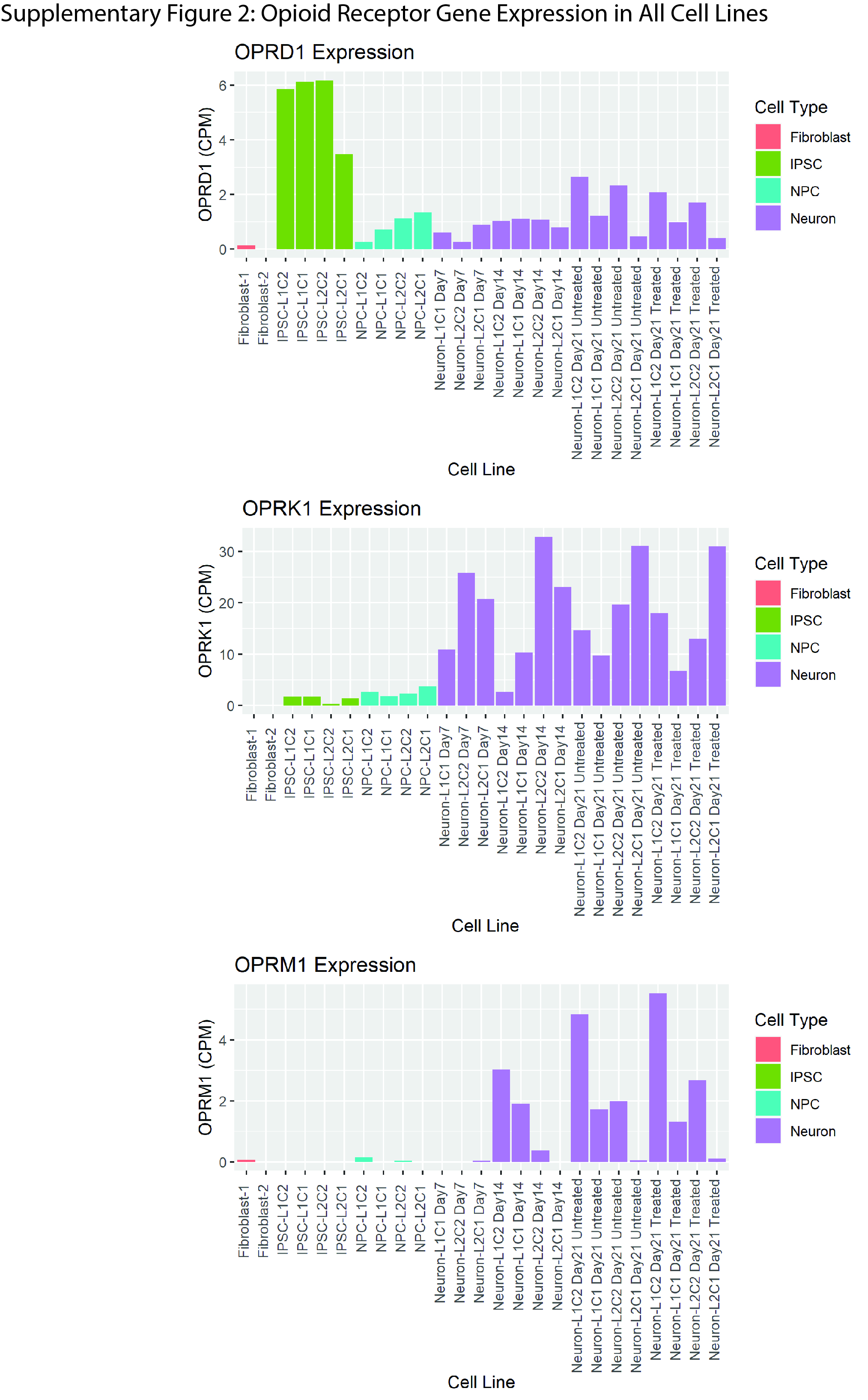

Supplement: Supplementary file 13 [file Image_2.JPEG]

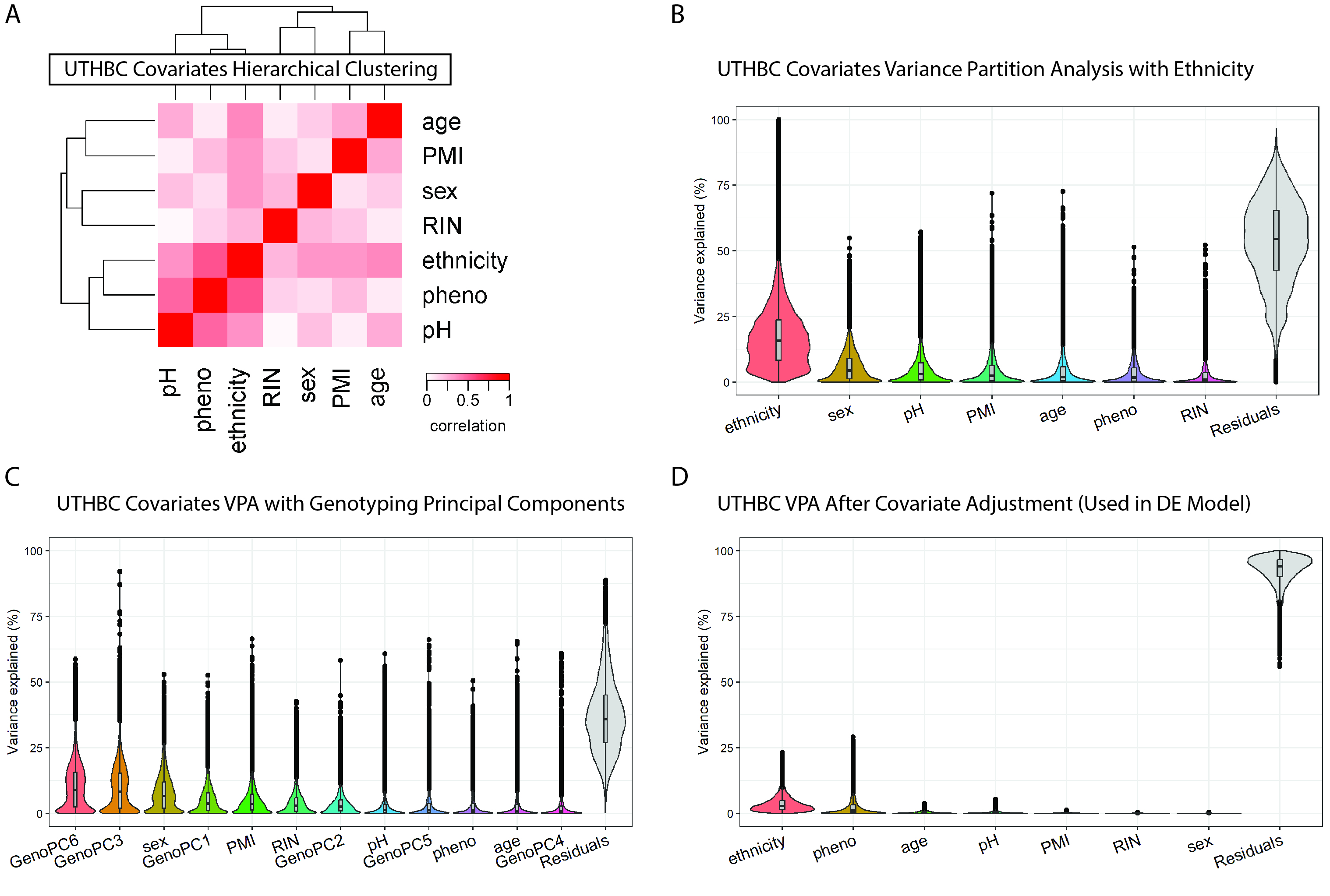

Supplement: Supplementary file 14 [file Image_3.JPEG]

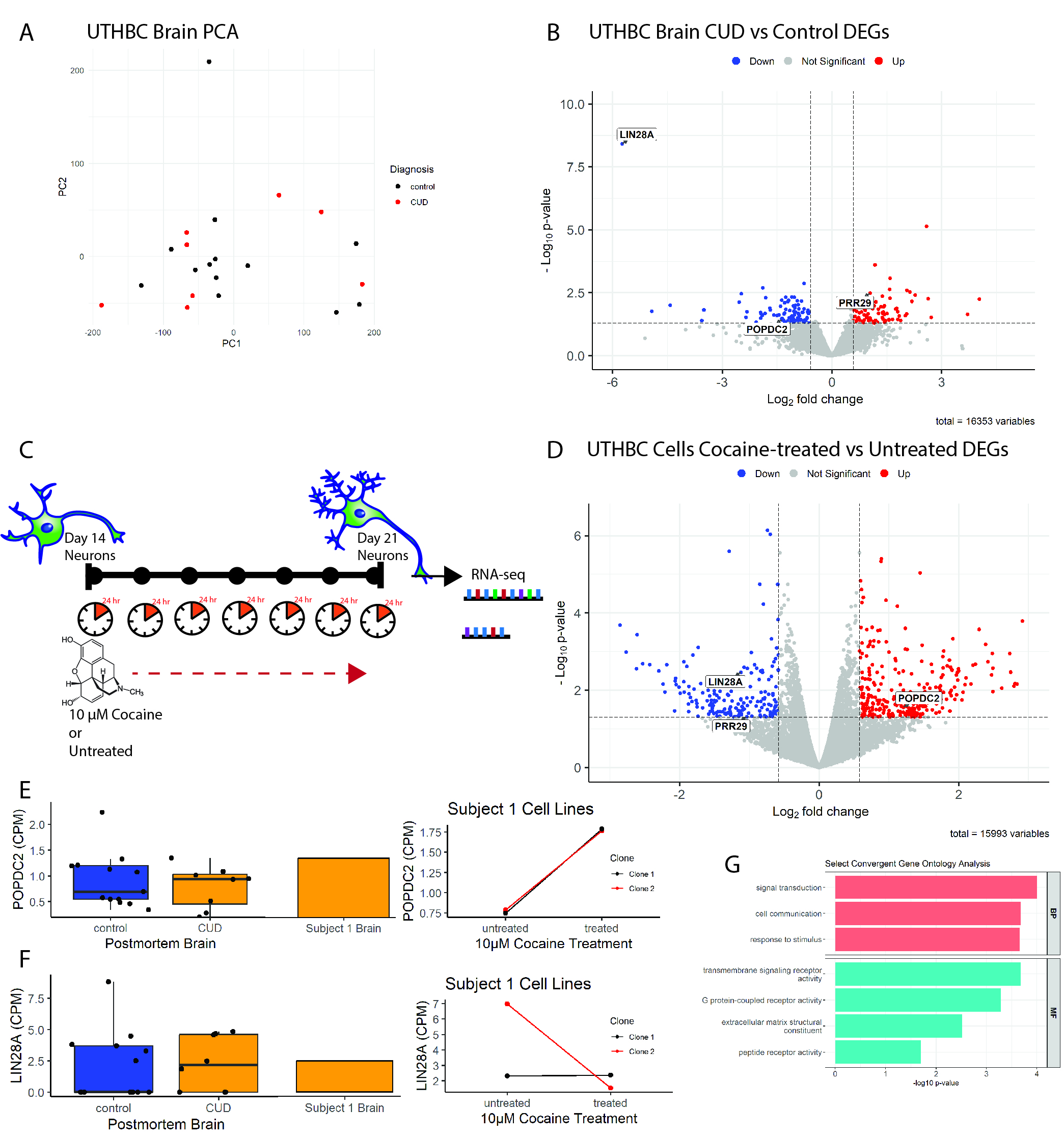

Supplement: Supplementary file 15 [file Image_4.JPEG]
